# Supplementary material for: End Sequence Analysis Toolkit (ESAT) expands the extractable information from single-cell RNA-seq data
Source: Genome Res. 2016 Oct;26(10):1397–410. doi: 10.1101/gr.207902.116 (PMC5052061; doi:10.1101/gr.207902.116)
Supplement: Supplemental Material [file supp_gr.207902.116_Supp_Materials.tar.gz › ESAT-esat_v0.1/doc/html/classumms_1_1core_1_1general_1_1_predicates.html]

ESAT: umms.core.general.Predicates Class Reference


ESAT


- Main Page
- Related Pages
- Packages
- Classes
- Files

- Class List
- Class Index
- Class Hierarchy
- Class Members

All Classes Namespaces Files Functions Variables Enumerator Pages

- umms
- core
- general
- Predicates

Classes |
Static Public Member Functions |
List of all members

umms.core.general.Predicates Class Reference

|  |  |
| --- | --- |
| Static Public Member Functions | |
| static< T > Predicate< T > | alwaysTrue () |
|  | |
| static< T > Predicate< T > | alwaysFalse () |
|  | |
| static< T > Predicate< T > | and (Iterable<?extends Predicate<?super T >> components) |
|  | |
| static< T > Predicate< T > | and (Predicate<?super T >...components) |
|  | |
| static< T > Predicate< T > | not (Predicate< T > predicate) |
|  | |
| static< T > Predicate< T > | or (Iterable<?extends Predicate<?super T >> components) |
|  | |
| static< T > Predicate< T > | or (Predicate<?super T >...components) |
|  | |

## Detailed Description

Author
:   engreitz Inspired by Google Guava Predicates class

## Member Function Documentation

|  |  |  |  |  |
| --- | --- | --- | --- | --- |
| static <T> Predicate<T> umms.core.general.Predicates.alwaysFalse | ( |  | ) |  |

static

|  |  |  |  |  |
| --- | --- | --- | --- | --- |
| static <T> Predicate<T> umms.core.general.Predicates.alwaysTrue | ( |  | ) |  |

static

Here is the caller graph for this function:

|  |  |  |  |  |  |
| --- | --- | --- | --- | --- | --- |
| static <T> Predicate<T> umms.core.general.Predicates.and | ( | Iterable<?extends Predicate<?super T >> | *components* | ) |  |

static

Returns a predicate that evaluates to

true

if the object being tested passes all component predicates. If {} is empty, the predicate will always return true. It defensively copies the iterable passed in, so future changes to the iterable will not alter the behavior of this predicate.

Parameters
:   |  |  |
    | --- | --- |
    | <T> |  |
    | components | Collection of predicates to evaluate |

Returns

Here is the caller graph for this function:

|  |  |  |  |  |  |
| --- | --- | --- | --- | --- | --- |
| static <T> Predicate<T> umms.core.general.Predicates.and | ( | Predicate<?super T >... | *components* | ) |  |

static

|  |  |  |  |  |  |
| --- | --- | --- | --- | --- | --- |
| static <T> Predicate<T> umms.core.general.Predicates.not | ( | Predicate< T > | *predicate* | ) |  |

static

|  |  |  |  |  |  |
| --- | --- | --- | --- | --- | --- |
| static <T> Predicate<T> umms.core.general.Predicates.or | ( | Iterable<?extends Predicate<?super T >> | *components* | ) |  |

static

|  |  |  |  |  |  |
| --- | --- | --- | --- | --- | --- |
| static <T> Predicate<T> umms.core.general.Predicates.or | ( | Predicate<?super T >... | *components* | ) |  |

static

---

The documentation for this class was generated from the following file:

- java/umms/core/general/Predicates.java


---

Generated on Fri Aug 15 2014 15:11:11 for ESAT by  

 1.8.7
